# Comprehensive analysis of the LINC01122/TPD52 axis as a predictive biomarker in prostate adenocarcinoma

**DOI:** 10.1038/s41598-025-98219-1

**Published:** 2025-05-08

**Authors:** Zhi Shang, Shengming Jin, Yandong He, Yiping Zhu, Hailiang Zhang, Junlong Wu, Zhe Hong, Dingwei Ye

**Affiliations:** 1 Department of Urology, Fudan University Shanghai Cancer Center, Shanghai, 200032 China; 2 Department of Oncology, Shanghai Medical College, Fudan University, Shanghai, 200032 China; 3 Shanghai Genitourinary Cancer Institute, Shanghai, 200032 China; 4 Department of Urology, South Hospital, The Sixth People’s Hospital of Shanghai, Shanghai, 201499 China

**Keywords:** Prostate adenocarcinoma, ceRNA network, Prognosis, LINC01122/TPD52 axis, Cancer, Biomarkers, Urology

## Abstract

Prostate cancer (PCa) ranks among the most prevalent malignant tumors worldwide. The pivotal role of competitive endogenous RNA (ceRNA) regulatory networks in numerous cancer types has been underscored. However, the specific characteristics of the ceRNA network in PCa remained unknown. This study aims to elucidate the ceRNA regulatory network associated with phosphatase and tensin homolog (PTEN) and to identify potential prognostic markers for PCa. The Cancer Genome Atlas (TCGA) database was employed to extract the expression patterns of long non-coding RNAs (lncRNAs), microRNAs (miRNAs), and messenger RNAs (mRNAs). LINC01122-hsa-miR-34c-5p/hsa-miR-449a-TPD52 ceRNA network regarding the prognosis of PCa was explored via bioinformatics analysis. Through correlation analysis, we investigated the LINC01122/TPD52 axis within the ceRNA network, identifying it as a significant clinical prognostic marker for PCa. Subsequent analyses indicated that hypomethylation was responsible for the abnormal upregulation of the LINC01122/TPD52 axis. Furthermore, immune infiltration analysis revealed the impact of the LINC01122/TPD52 axis on the tumor immune microenvironment and the progression of PCa. Finally, a nomogram was constructed to forecast the 1-year, 3-year, and 5-year survival probabilities of PCa patients. In summary, our study demonstrates the significant role of the ceRNA-based LINC01122/TPD52 axis in the progression of PCa and its correlation with prognosis.

## Introduction

Prostate cancer (PCa) is one of the most common malignant tumors of the urological system in elderly men, with its incidence rate ranking the second highest among malignant tumors in men worldwide, and its mortality rate ranking the fifth highest among the causes of cancer deaths in men globally^1,2^. Prostate adenocarcinoma (PRAD), account for 95% of all PCa^3^. Although androgen deprivation therapy (ADT) is one of the main treatments for advanced PCa, patient prognosis remains poor, and treatment options for PRAD are limited^4^. Therefore, to create efficient treatment strategies and improve the prognosis of PRAD patients, the hunt for novel prognostic indicators is crucial.

The Cancer Genome Atlas (TCGA) is a well-known source of publicly accessible large-scale cancer omics data that gives us clinical and molecular details on a variety of cancer patients, which may be helpful in our search for candidate tumor biomarkers^5^. The increasing utilization of high-throughput RNA sequencing (RNA-seq) has driven the development of more efficient, non-invasive, repeatable, and accurate tools for patient monitoring and early screening.

Long noncoding RNAs (lncRNAs) are transcripts exceeding 200 nucleotides in length and are not translated into proteins^6^. LncRNAs wield substantial influence on the progression of various cancer types (including PRAD), and their expression is strongly correlated with tumorigenesis, metastasis, and poor prognosis^7,8^. As a result, the identification of specific lncRNA biomarkers associated with the prognosis and diagnosis of PRAD is considered of paramount clinical significance.

MicroRNAs (miRNAs) are short RNA molecules, typically 19 to 25 nucleotides in length, that inhibit gene degradation or translation by binding to the 3′ noncoding regions of their target mRNAs^9,10^. MiRNA expression patterns can be associated with cancer type, stage, and other clinical characteristics, making miRNA profiling an essential tool for cancer diagnosis and prognosis^11^. According to the competing endogenous RNAs (ceRNAs) hypothesis, the researchers considered that lncRNA could indirectly regulate mRNA expression by competing with miRNA as a natural sponge in the cytoplasm, which would eventually result in the weakness of interaction between miRNAs and mRNAs^12,13^.

Phosphatase and tensin homolog deleted on chromosome ten (PTEN) is a tumor suppressor that exhibits widespread expression in human malignancies, including PRAD^3,14^. PTEN activity can be regulated through mutations, epigenetic silencing, aberrant protein, transcriptional inhibition, location, and posttranslational modification^15,16^. Simultaneously, the loss of PTEN tumor suppressor function is recognized as one of the most frequent driving events in PRAD development^17^.

**Fig. 1:**
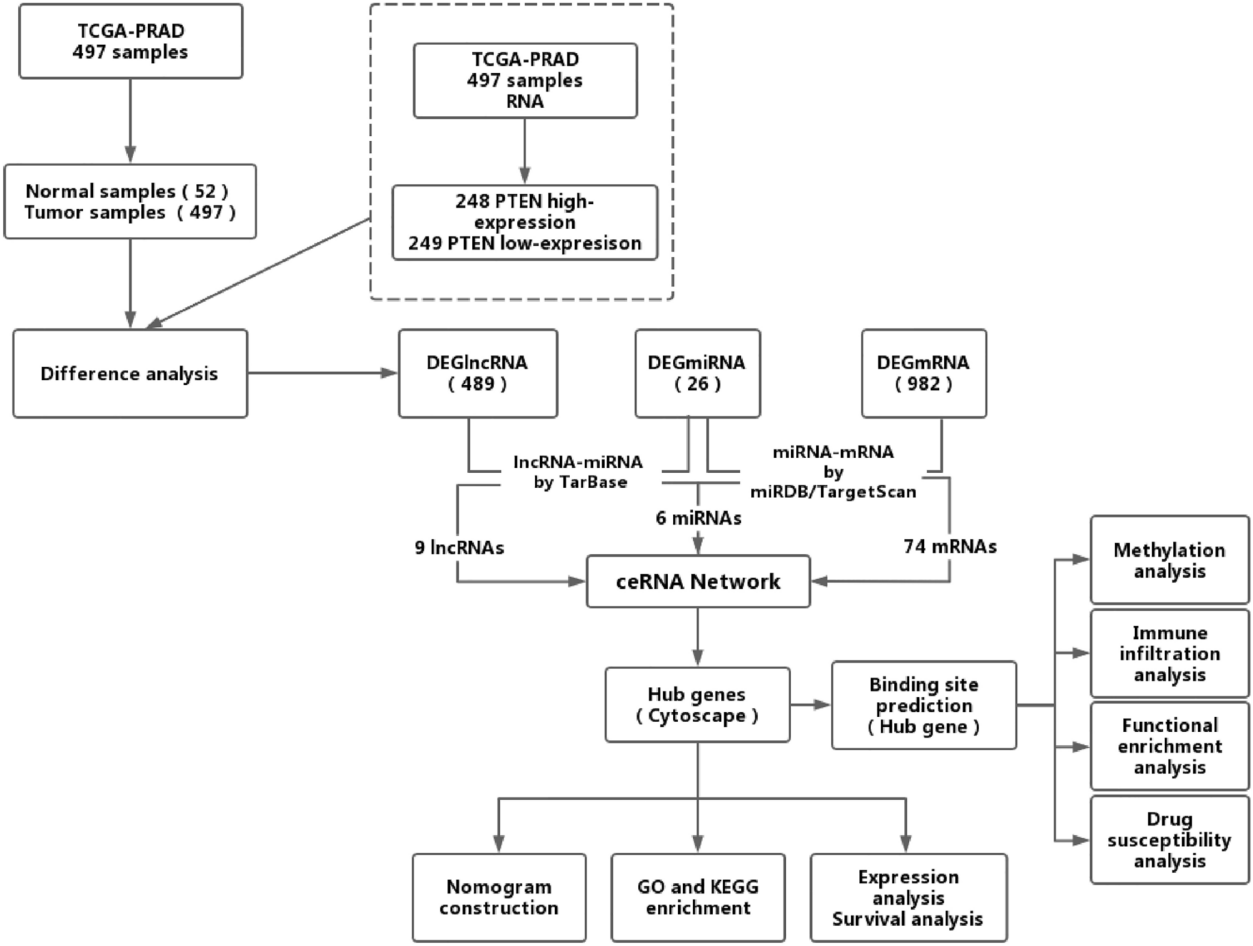
Flowchart of construction and analysis of ceRNA.

In this study, we constructed a ceRNA network associated with PTEN in in PRAD (Fig. 1). The PRAD related lncRNA-miRNA-mRNA triple regulatory networks were built from three differently expressed RNAs using differential expression analysis in two groups of PTEN^high^ and PTEN^low^ expression in 497 PRAD samples. The expression, survival, and nuclear-cytoplasmic localization analysis of RNAs from hub triple regulatory networks were used to identify a critical ceRNA network. Furthermore, our analysis between this predicted gene and PTEN reveals that the LINC01122/TPD52 axis is significant in PRAD. We employed Cox regression analysis to assess the diagnostic and prognostic value of TPD52 in PRAD. To ascertain the potential functions of TPD52 in PRAD, we conducted gene ontology (GO) and Kyoto encyclopedia of genes and genomes (KEGG) analyses^18^. Additionally, we performed methylation analysis, immune infiltration analysis, functional enrichment analysis, and drug susceptibility analysis to further investigate the biological functions of TPD52 in PRAD.

## Materials and methods

### Data collection and processing

We retrieved RNAseq data from the TCGA database using the R software TCGAbiolinks package. We acquired 497 PRAD samples (including 52 paired PRAD samples) available mRNA sequencing (mRNA-seq) data and 494 PRAD samples (including 52 paired PRAD samples) available miRNA-sequencing (miRNA-seq) data from the TCGA database (https://portal.gdc.cancer.gov/). We normalized the raw RNA-seq data (lncRNAs, miRNAs, and mRNAs) to fragments per kilobase of the exon model per million mapped fragment reads. We utilized the starBase version 2.0 database (http://starbase.sysu.edu.cn) to convert miRNA sequences to human mature miRNA names. Meanwhile, we validated the expression of PTEN and TPD52 at the protein level using HPA (http://www.proteinatlas.org/). The cBioPortal of Cancer Genomics (http://www.cbioportal.org/) was used to obtain the mutational status of PTEN.

### DElncRNAs, DEmiRNAs, and DEmRNAs in PRAD

In PRAD samples for differential expression analysis at PTEN^high^ and PTEN^low^, we identified DElncRNAs based on thresholds |logFC|> 0.7 and FDR < 0.05, DEmiRNAs based on thresholds of |logFC|> 0.5 and FDR < 0.05, and DEmRNAs based on thresholds of |logFC|> 0.7 and FDR < 0.05. In the differential expression analysis between PRAD samples and adjacent non-tumor samples, cutoff values were set as FDR < 0.05 and |logFC|> 0.5 for DElncRNAs, FDR < 0.05 and |logFC|> 0.5 for DEmiRNAs, and FDR < 0.05 and |logFC|> 0.7 for DEmRNAs. We used ggplot2 package and pheatmap package in R to generate volcano plots of the DERNAs (including DElncRNAs, DEmiRNAs, and DEmRNAs) and the heatmap clustering respectively.

### Construction of ceRNA regulatory network in PRAD

We utilized TarBase (TarBase v8.0 database, http://microrna.gr/tarbase/) to forecast potential miRNAs targeted by DElncRNAs as well as lncRNA-miRNA interaction pairs. For the prediction of DEmiRNA target genes and the construction of miRNA-mRNA interaction pairs, we utilized MiRWalk (http://mirwalk.umm.uni-heidelberg.de/). We employed the VennDiagram package in R to compare the target genes with DEmRNAs and chose the target genes that overlapped with DEmRNAs for the next step of the analysis. The ceRNA regulatory network of lncRNA-miRNA-mRNA was established by incorporating the lncRNA-miRNA pairs with miRNA-mRNA pairs. We used LNcipedia (https://lncipedia.org/) and the lncLocator (http://www.csbio.sjtu.edu.cn/bioinf/lncLocator/) database to acquire DElncRNA sequences and identify DElncRNA cellular localization according to its sequence. Cytoscape software (version 3.9.1, https://www.cytoscape.org/) was used to visualize the ceRNA network.

### Functional enrichment analysis

To apprehend the possible biological processes and pathways of the network, we performed the functional enrichment analysis of DEmRNAs in the triple regulatory network of lncRNA-miRNA-mRNA in Metascape (http://metascape.org/gp/index.html).

### Survival analysis and construction of a specific prognosis model for PRAD

Kaplan–Meier analysis and the log-rank test were performed for DElncRNA, DEmiRNA, and DEmRNA within the ceRNA network using R software to assess their association with PFS in PRAD patients from the TCGA database. To identify biomarkers associated with prognosis, univariate Cox regression analysis was employed to examine the relationship between candidate genes in the ceRNA network and PFS. P-values were calculated for each hypothesis test, and a threshold of < 0.05 was initially considered statistically significant. To account for multiple hypothesis testing, we applied False Discovery Rate (FDR) correction to control for false positives. Only results with an FDR-adjusted p-value < 0.05 were considered statistically significant.

### Methylation and expression analysis of TPD52

Three DNA methyltransferases (DNMT1, DNMT3A, and DNMT3B), according to studies, could control gene expression by DNA methylation and have an impact on the behavior of cancer cells. Initially, we investigated the expression level of three DNA methyltransferases in TPD52^high^ and TPD52^low^ groups by TCGA database. Then we utilized UALCAN (http://ualcan.path.uab.edu/) to assess the relationship between methylation levels of TPD52 in PRAD and adjacent normal tissues. Furthermore, we used MEXPRESS (https://mexpress.be) to investigate the association between the expression of the central gene and its DNA methylation status.

### Immune infiltrate level and expression analysis of TPD52

We utilized TIMER (https://cistrome.shinyapps.io/timer/), a tool for assessing and visualizing immune infiltrates across various cancer types, to explore the relationship between TPD52 expression and tumor-infiltrating immune cells. We investigated the association between TPD52 expression, tumor-infiltrating immune cells (including B cells, CD4 + T cells, CD8 + T cells, neutrophils, macrophages, and dendritic cells), prognostic value, and the correlation between TPD52 gene copy number and PRAD.

### Construction and evaluation of nomogram

We constructed a nomogram with the “rms” R package to show the relationship between the variables and the prognostic model and we used 1-, 3-, and 5-year calibration curves to identify and predict the value.

### Drug sensitivity analysis

We employed the pRRophetic package in R software to calculate sensitivity scores for each small molecule compound for both high-risk and low-risk patient groups.

### Statistical analysis

We performed data analysis using R software (version 4.1.1). The results are presented as medians with accompanying 95% confidence intervals (CI). To evaluate differences between the two groups, we applied the rank sum test. For comparisons between multiple groups, we utilized one-way ANOVA with the Kruskal–Wallis test and the chi-square test. P < 0.05 was considered as a statistically significant difference.

## Results

### The significance of PTEN overexpression in PRAD for prognosis and its tumor suppressor role

**Fig. 2:**
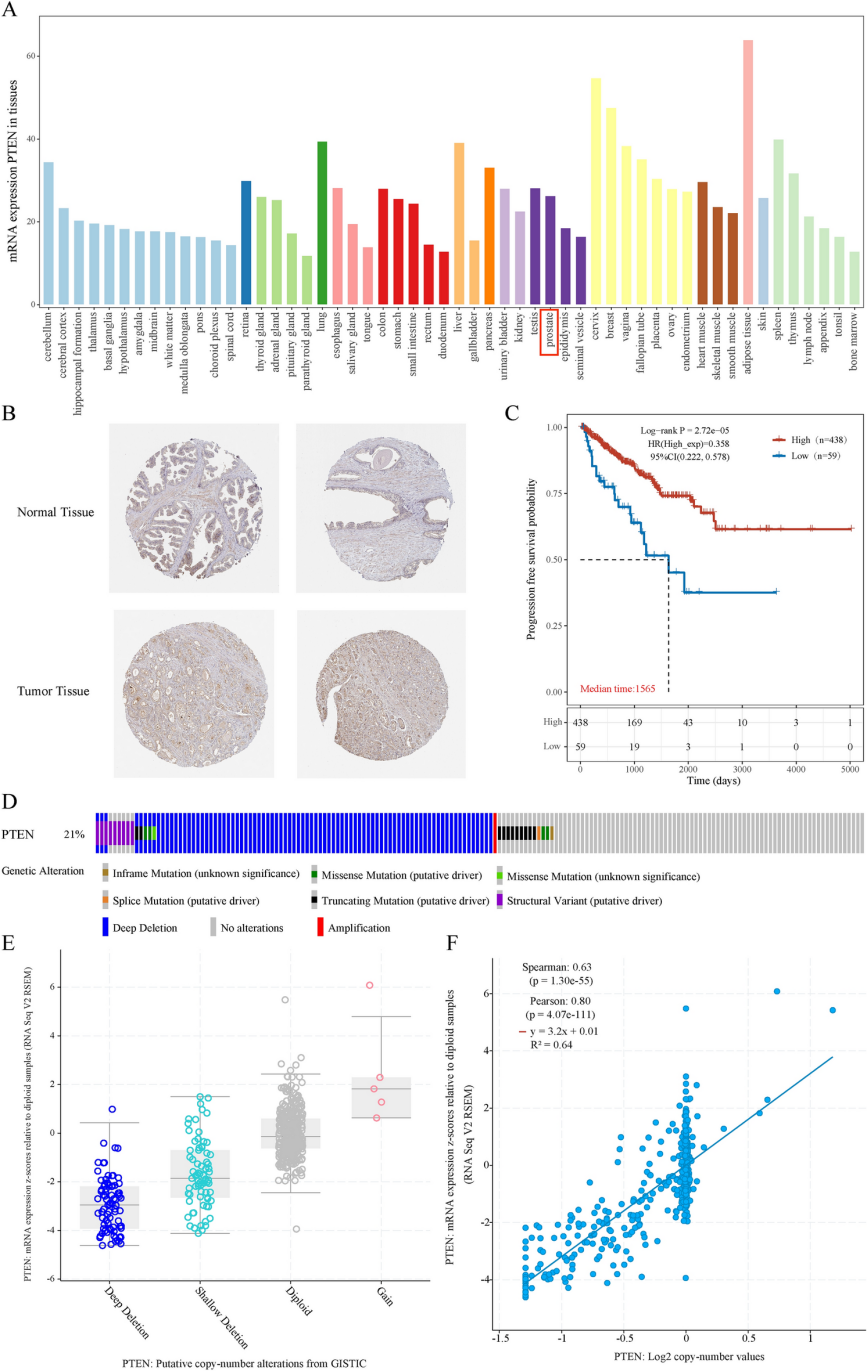
The role of PTEN as a tumor suppressor in human prostate cell carcinoma (PRAD). (**A**) Expression distribution of PTEN in pan-cancer tissues. (**B**) Verification of the expression of PTEN on the translational level by the Human Protein Atlas database (immunohistochemistry). (**C**) The Kaplan–Meier survival curve was used to compare the low (n = 59) and high (n = 438) expression of PTEN. (**D**) The distribution of PTEN genomic alterations in TCGA PRAD is shown on a cBioPortal OncoPrint plot. (**E**,**F**) The association between PTEN copy number and mRNA expression are shown in the dot plot (**E**) and correlation plot (**F**) by cBioPortal.

We found that PTEN is overexpressed in normal prostate tissue but downregulated in PRAD tissue in the Human Protein Atlas (HPA) database (Fig. 2A). PTEN expression dysregulation was also seen in HPA immunohistochemical (IHC) staining (Fig. 2B). As PTEN was abnormally downregulated in tumor specimens, we subsequently investigated the clinical significance of PTEN expression in patients with PRAD. Our data suggested that lower expression of PTEN significantly correlated with poor progression-free survival (PFS) in patients of the PRAD cohort, as illustrated by the Kaplan–Meier survival curves in (Fig. 2C). And we analyzed the genomic and copy number of PTEN to comprehend the underlying mechanism of the abnormally low expression of PTEN in PRAD. The OncoPrint depicted the deletion of the PTEN gene in the TCGA PRAD dataset, consistent with previous studies on PTEN loss in prostate cancer^3^, as a consequence of our investigation on cBioPortal, which yielded the following results (Fig. 2D). As is shown in (Fig. 2E), more than one-fourth of all PRAD samples contained PTEN loss, and PRAD samples containing a PTEN deletion displayed lower mRNA expression than those with diploid PTEN. Additionally, PRAD samples revealed a positive correlation between PTEN copy number and mRNA expression (Fig. 2F). Together, these findings imply that PTEN expression is downregulated in PRAD and that one of the key mechanisms contributing to PTEN downregulation in PRAD patients is the loss of PTEN copy number.

### Identification of differentially expressed genes (DEGs) (DEmRNAs, DElncRNAs, and DEmiRNAs)

**Fig. 3:**
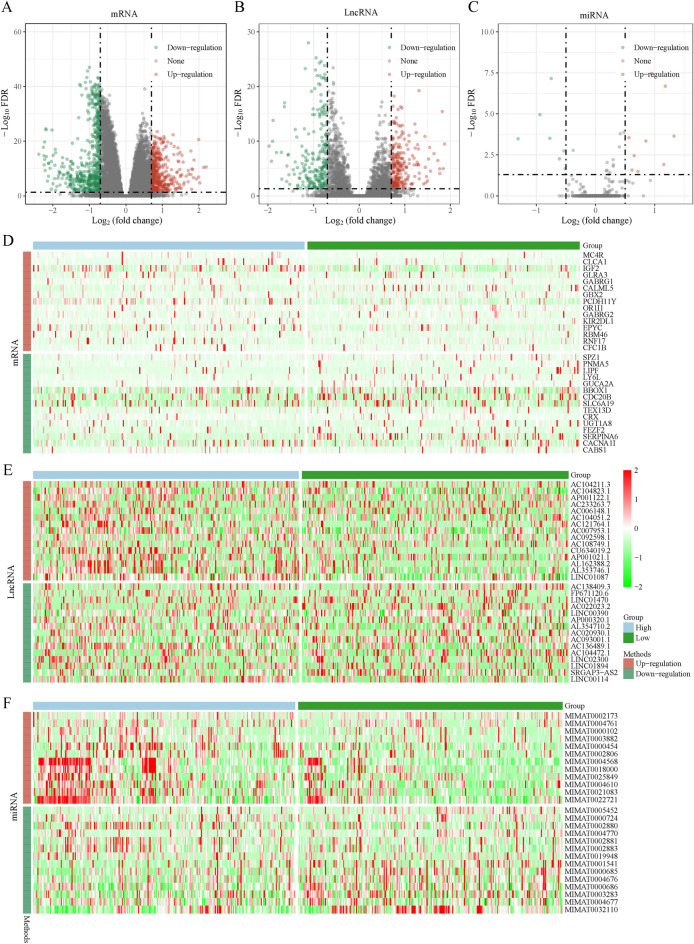
Volcano plots and heatmap plots of DEmRNAs, DElncRNAs and DEmiRNAs between the expression of PTEN^high^ and PTEN^low^ in PRAD samples. Red represents upregulated genes and green indicates downregulated genes. (**A**–**C**) The volcano plots describe (**A**) 982 DEmRNAs (FDR < 0.05 and |logFC|> 0.7) (**B**) 489 DElncRNAs (FDR < 0.05 and |logFC|> 0.7), and (**C**) 26 DEmiRNAs (FDR < 0.05 and |logFC|> 0.5). (**D**–**F**) The horizontal axis of the heatmap indicates the samples, and the vertical axis of the heatmap indicates 30 significant DEGs.

The ceRNA network linked to PTEN, as determined by the studies above, may function as a potential prognostic model for PRAD patients. We need to clarify the meaning of the expression levels of PTEN^high^ expression groups and PTEN^low^ expression groups in PRAD samples are opposite to those in cancer and paracancerous groups. To begin, we used the TCGA database to identify the DElncRNAs, DEmiRNAs, and DEmRNAs in PRAD samples with PTEN^high^ and PTEN^low^ expression groups as well as in PRAD and nearby normal tissues, with FDR < 0.05 and |logFC|> 0.7 as lncRNA thresholds, FDR < 0.05 and |logFC|> 0.5 as miRNA thresholds, and FDR < 0.05 and |logFC|> 0.7 as mRNA thresholds. A total of 489 DElncRNAs (248 upregulated and 241 downregulated), 26 DEmiRNAs (12 upregulated and 14 downregulated), and 982 DEmRNAs (410 upregulated and 572 downregulated) were screened from the PRAD samples with PTEN^high^ and PTEN^low^ expression groups. In addition, 2398 DElncRNAs (1177 upregulated and 1221 downregulated), 268 DEmiRNAs (146 upregulated and 122 downregulated), and 3816 DEmRNAs (1568 upregulated and 2248 downregulated) were identified between PRAD samples and normal prostate tissue samples. Visualizations of the distribution of DElncRNAs, DEmiRNAs, and DEmRNAs were created using volcano plots (Fig. 3A–C and Fig. S1A–C), and the heatmaps show the expression of 30 significant variable genes in PRAD samples with PTEN^high^ and PTEN^low^ expression, as well as in PRAD and normal samples (Fig. 3D–F and Fig. S1D–F).

### Construction of the lncRNA-miRNA-mRNA triple regulatory network

**Fig. 4:**
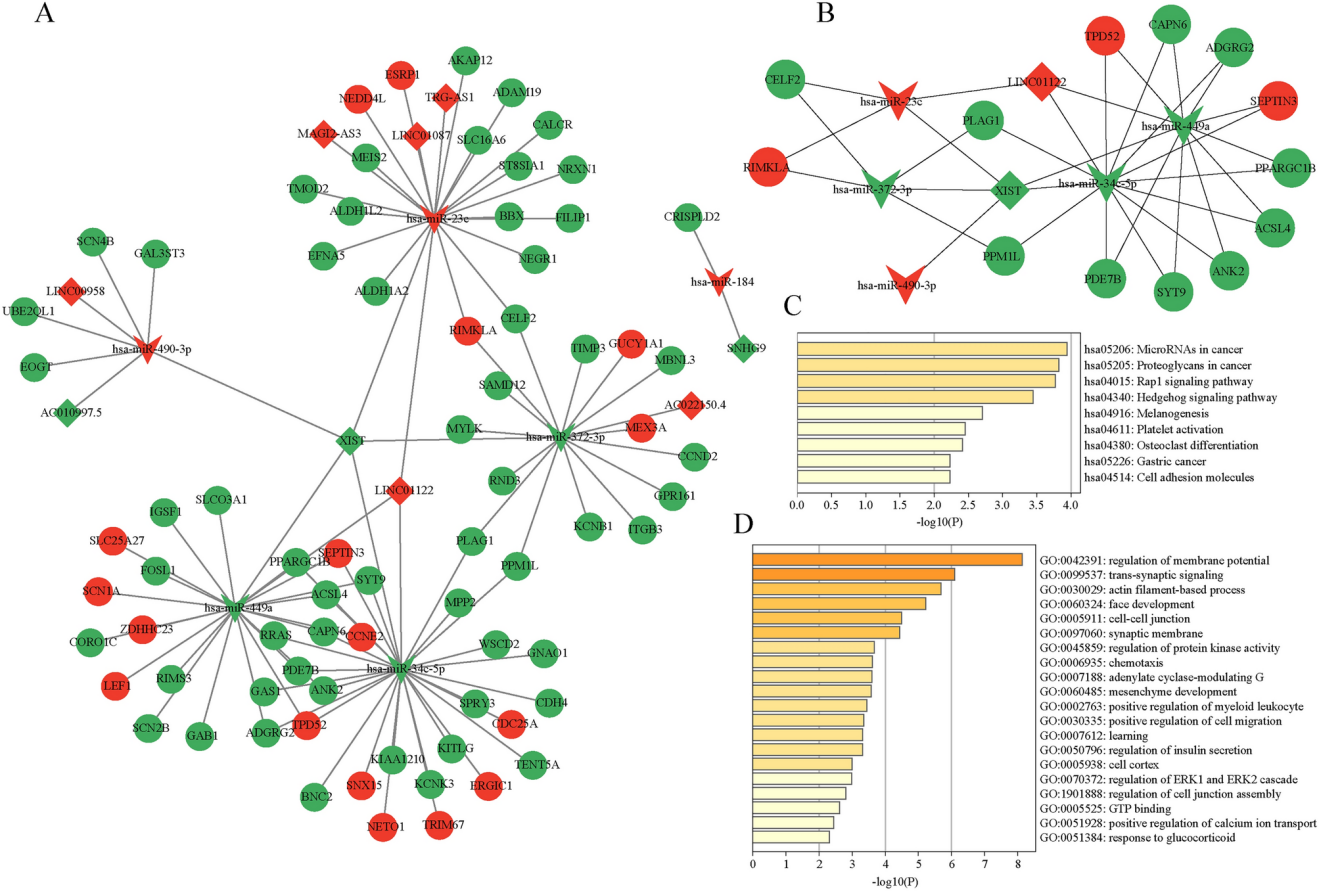
The construction and functional enrichment analysis of the lnRNA-miRNA-mRNA triple regulatory network. Inverted triangles represent miRNAs, diamonds represent lncRNAs, and circles represent mRNAs. (**A**) The triple regulatory network in PRAD. Red indicates upregulated, and green represents downregulated. (**B**) Twenty hub genes in this network with a score of > 2. (**C**) Functional enrichment analysis (GO and KEGG) of the DEmRNAs in the network.

We performed a joint analysis in the PTEN^high^ and PTEN^low^ expression groups as well as in PRAD and normal prostate tissue groups. To distinguish potential miRNAs targeting lncRNAs, we put DElncRNAs into the TarBase database. Then We utilized the databases of miRDB and TargetScan to identify downstream target mRNAs. Furthermore, we sought candidate mRNAs that were shared only by the two databases to improve the accuracy of predictions. Ultimately, 9 lncRNAs (6 upregulated and 3 downregulated), 6 miRNAs (3 upregulated and 3 downregulated), and 74 mRNAs (17 upregulated and 57 downregulated) were made up of the PRAD-associated lncRNA-miRNA-mRNA triple regulatory network by using Cytoscape software (Fig. 4A).

The hub triple regulatory network was identified using the Cytoscape plug-in cytoHubba. The results revealed that two lncRNAs (XIST and LINC01122), five miRNAs (hsa-miR-34c-5p, hsa-miR-23c, hsa-miR-372-3p, hsa-miR-449a and hsa-miR-490-3p), and thirteen mRNAs (RIMKLA, CELF2, TPD52, PLAG1, ACSL4, PPARGC1B, PPM1L, SEPTIN3, SYT9, ANK2, CAPN6, PDE7B and ADGRG2) were identified (Fig. 4B). Functional enrichment analysis (including GO and KEGG) was used by Metascape to further investigate the potential roles connected to the triple regulatory network. The findings demonstrated that the network’s DEmRNAs were notably enriched in the “proteoglycans in cancer,” “Rap1 signaling pathway,” and “GTP binding” pathways (Fig. 4C,D).

### Construction of ceRNA network and model with PRAD-specific prognostic value

**Fig. 5:**
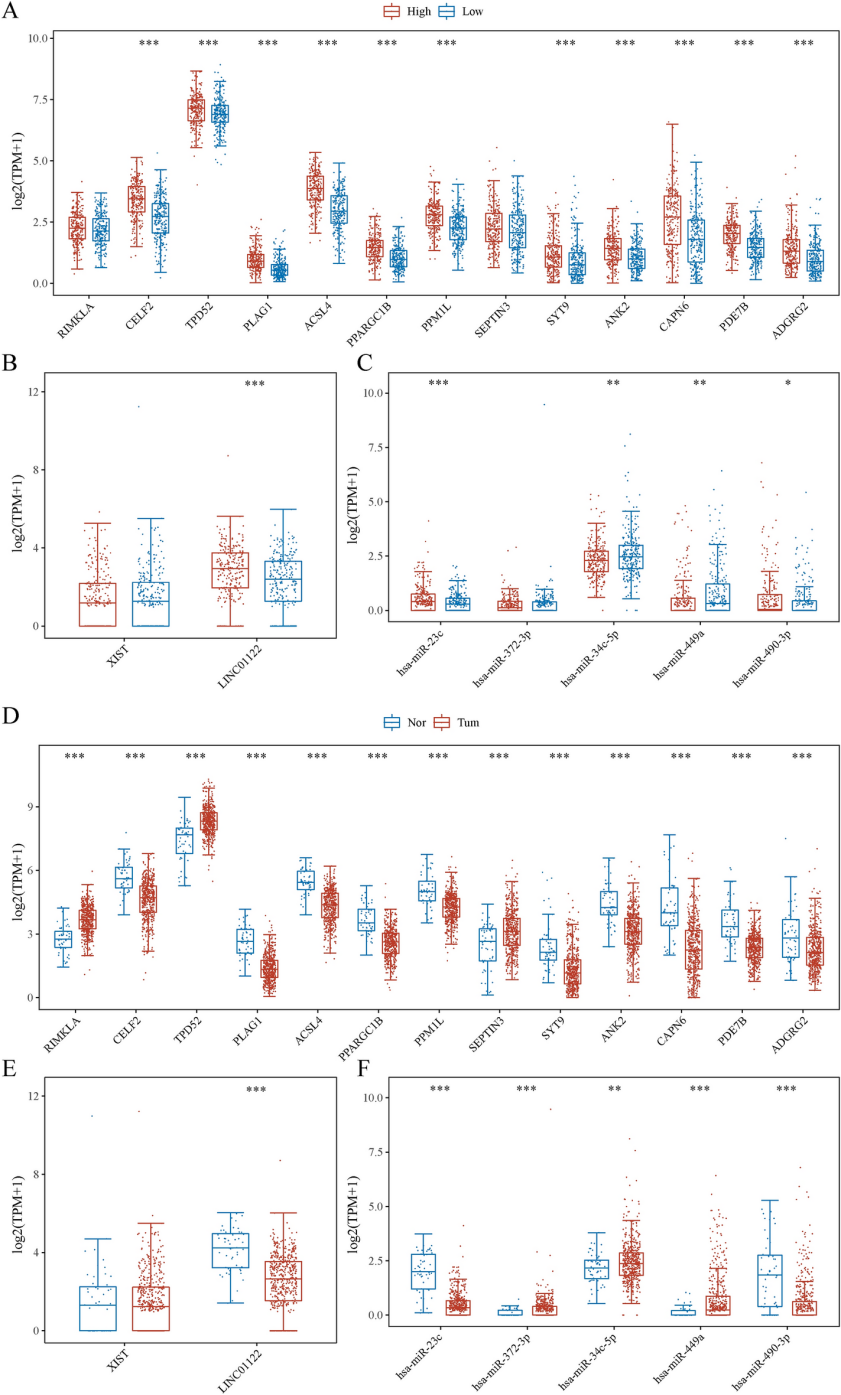
The distribution of 20 hub-RNA expression patterns from the triple regulatory network in TCGA PRAD dataset. (**A**–**F**) The expression patterns of 13 hub-DEmRNAs, two hub-DElncRNAs, and five hub-DEmiRNAs in PRAD samples with PTENhigh and PTENlow expression groups (**A**–**C**), and in PRAD and adjacent normal liver tissues (**D**–**F**).

To identify a critical ceRNA with significant prognostic significance in PRAD, we first examined the expression levels of RNAs from the hub triple regulatory network in PRAD samples with PTEN^high^ and PTEN^low^ expression groups and in PRAD and adjacent normal prostate tissue. The hub node in PTEN^high^ and PTEN^low^ expression groups were shown in Fig. 5A–C, CELF2, TPD52, PLAG1, ACSL4, PPARGC1B, PPM1L, SYT9, ANK2, CAPN6, PDE7B, and ADGRG2 were found to be significantly overexpressed in the high expression group in (Fig. 5A), while other genes were not significantly. LINC01122 was overexpressed in the high expression group in (Fig. 5B) and hsa-miR-23c and hsa-miR-490-3p were significantly highly expressed in the high expression group, and hsa-miR-34c-5p and hsa-miR-449a were significantly low expressed in the high expression group (Fig. 5C). The hub node in PRAD and adjacent normal prostate tissues was shown in (Fig. 5D–F), RIMKLA, TPD52, and SEPTIN3 were highly expressed in PRAD, while other genes were low-expressed in tumor tissues in (Fig. 5D). LINC01122 was low expressed in Fig. 5E and hsa-miR-372-3p, hsa-miR-34c-5p and hsa-miR-449a were overexpressed, while hsa-miR-23c and hsa-miR-490-3p were low expressed in PRAD in (Fig. 5F).

Then, to ascertain whether these RNAs were related to PRAD prognosis, we employed the Kaplan–Meier analysis and the log-rank test to perform PFS analysis of PRAD patients. In total, two DElncRNAs (XIST and LINC01122), four miRNAs (hsa-miR-490-3p, hsa-miR-449a, hsa-miR-23c, and hsa-miR-34c-5p), and eight DEmRNAs (RIMKLA, CELF2, TPD52, PLAG1, PPM1L, ANK2, CAPN6, and ADGRG2) were found to be associated with prognosis according to p < 0.05 (Fig. S2).

**Fig. 6:**
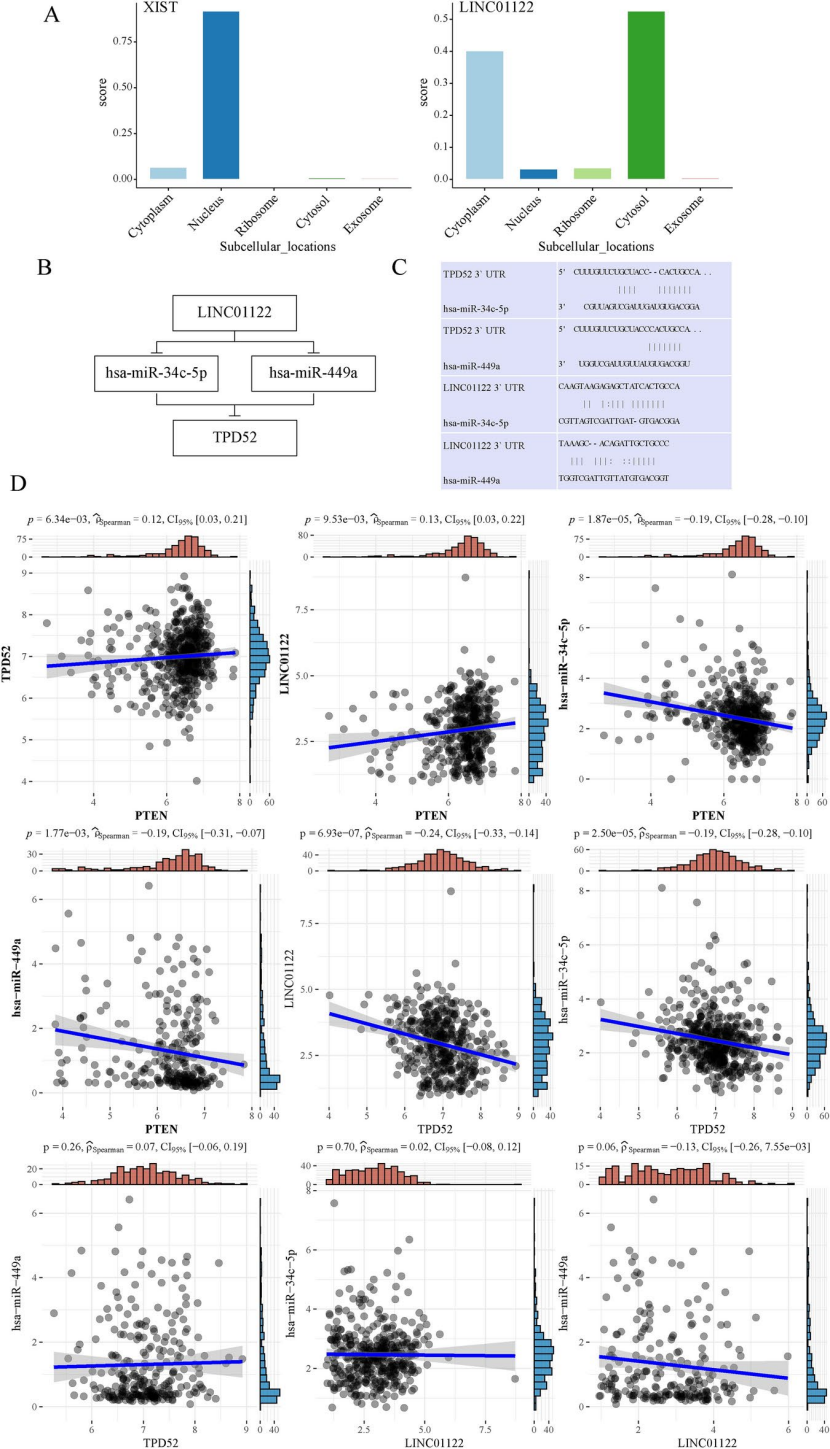
Construction and correlation analysis of the ceRNA network (**A**) The cellular localization for two hub-lncRNAs (LINC01122, and XIST) was predicted using lncLocator. (**B**) Schematic model of ceRNA. (**C**) Base pairing between hsa-miR-34c-5p and hsa-miR-449a and the target site in the LINC01122 and TPD52 3’ UTR predicted by TarBase and TargetScan, respectively. (**D**) Correlation analysis between these four predictive RNAs and PTEN in PRAD.

We also utilized the LncLocator to examine the subcellular localization of the two DElncRNAs because the cellular location of lncRNAs determined the underlying processes. LINC01122 is mainly located in the cytoplasm, while XIST is mainly distributed in the nucleus (Fig. 6A). These findings imply that LINC01122 may function as a ceRNA by sponging hsa-miR-34c-5p/hsa-miR-449a to enhance the expression of TPD52. Consequently, we constructed a ceRNA network of LINC01122-hsa-miR-34c-5p/hsa-miR-449a-TPD52. (Fig. 6B). The target sites in the LINC01122 and TPD52 3’ UTRs were predicted to pair with hsa-miR-34c-5p and hsa-miR-449a by TarBase and TargetScan, respectively (Fig. 6C). The expression correlation among the genes is shown in (Fig. 6D).

### Connection between TPD52 expression and methylation

**Fig. 7:**
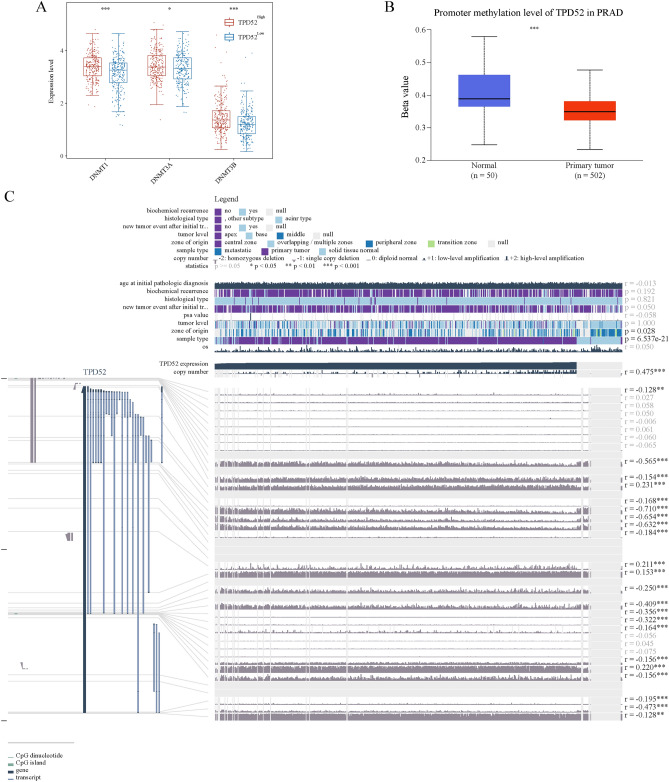
Methylation analysis of TPD52. (**A**) Differential expression of three DNA methyltransferases (DNMT1, DNMT3A, and DNMT3B). (**B**) Methylation was evaluated using UALCAN. (**C**) Visualization of methylation sites associated with gene expression in TPD52 DNA sequences using MEXPRESS.

To further illustrate the mechanism of aberrant TPD52 expression in PRAD tissues, we explored the correlation between TPD52 expression levels and its methylation status. Initially, differential expression analysis between TPD52^high^ and TPD52^low^ groups of PRAD revealed that the expression of the three DNA methyltransferases (DNMT1, DNMT3A, and DNMT3B) in the TPD52^high^ group was significantly higher than that in the TPD52^low^ group (Fig. 7A). In addition, the UALCAN analysis revealed that TPD52 has a tendency of higher methylation level in normal prostate tissues than in PRAD tissues (p < 0.0001, Fig. 7B). And we revealed 18 methylation sites in the DNA sequence of TPD52 that were negatively correlated with its expression level (Fig. 7C).

### Correlation between immune infiltration and expression of TPD52 in PRAD

**Fig. 8:**
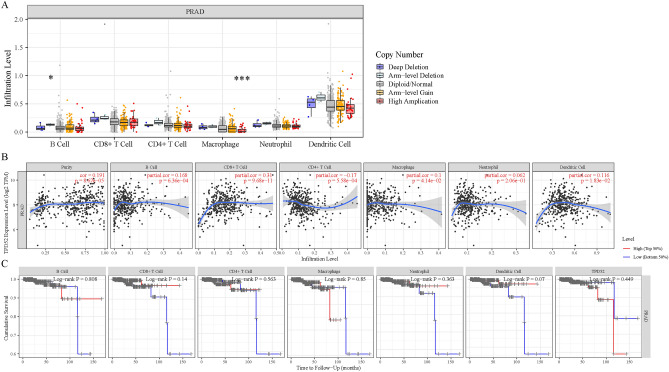
Correlation analysis of TPD52 expression and immune infiltration in PRAD. (**A**) Association between TPD52 gene copy number and immune cell infiltration levels in PRAD cohorts. (**B**) Correlation of TPD52 expression with immune infiltration level in PRAD. (**C**) Kaplan–Meier plots were used to analyze the immune infiltration and overall survival rate of PRAD. *p < 0.05, **p < 0.01, ***p < 0.001.

Tumor-infiltrating lymphocytes (TILs) have been reported to be independent predictors of sentinel lymph node (SLN) status and survival in certain cancers^19^. We utilized TIMER to perform the analysis below to assess the potential connection between TPD52 expression and immune infiltration levels in PRAD. First, the “SCNA” module analysis revealed that only B cells and macrophages showed significant differences, with the majority of immune cell infiltration levels not appearing to be related to changes in the copy number of the TPD52 gene (Fig. 8A). Then, the “genes” module analysis showed that TPD52 expression was correlated with tumor purity and B cells, and it is markedly positively correlated with infiltration levels of CD8 + T cells in PRAD (Fig. 8B). Finally, we further assessed the impact of immune infiltration on clinical outcomes in PRAD patients. The results suggest that the level of immune infiltration in PRAD patients is largely independent of poor prognosis (Fig. 8C).

### Independent prognostic analysis of pathological features and establishment of the nomogram

**Fig. 9:**
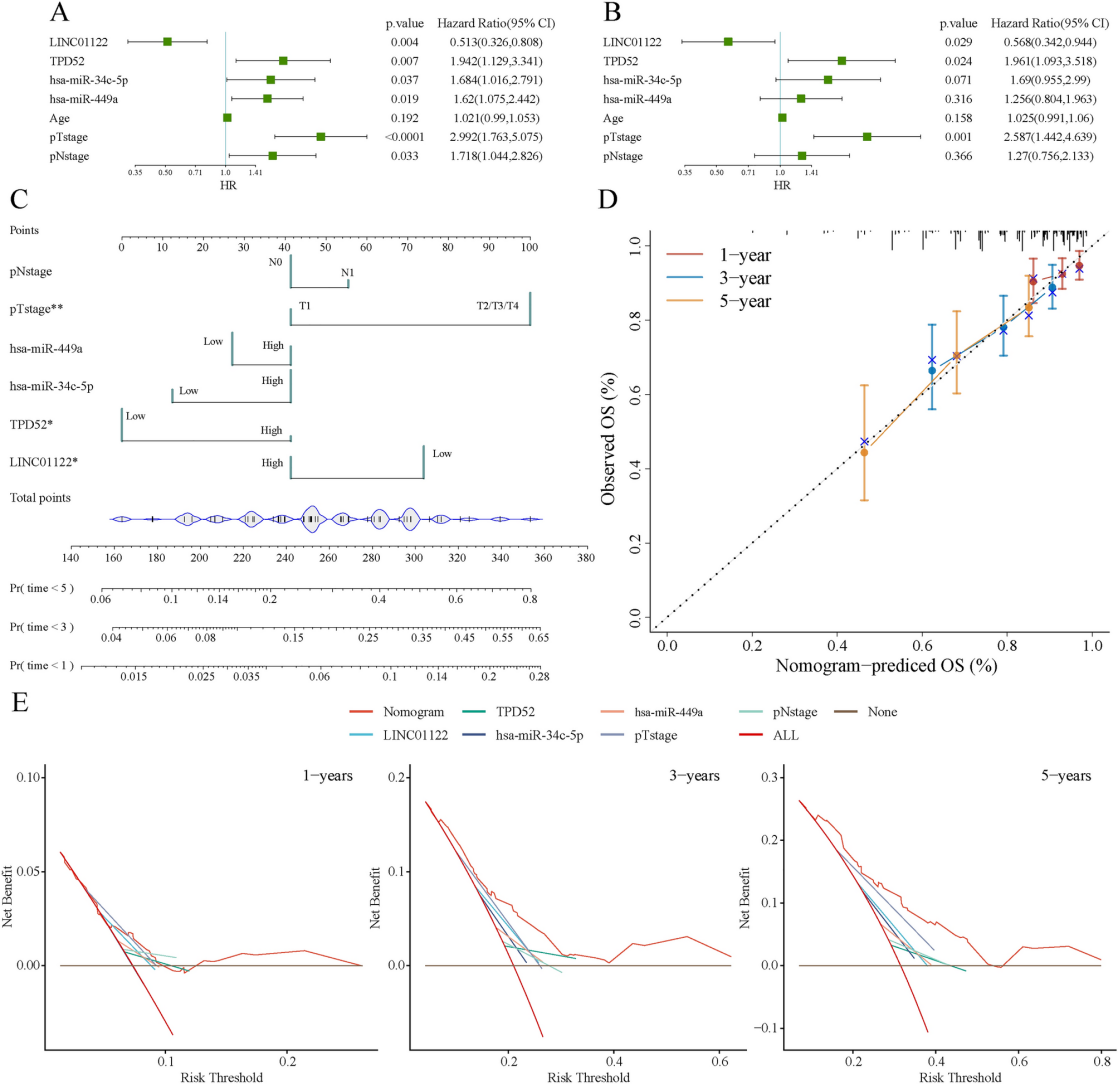
Independent prognostic analysis of pathological features and establishment of the nomogram. (**A**) Univariate Cox analysis of elements and clinical characteristics in ceRNA. (**B**) Multivariate Cox analysis of elements and clinical characteristics in ceRNA. (**C**) Establishment of the nomogram. (**D**) Establishment of the calibration curve. (**E**) The DCA decision curve of the nomogram in 1, 3, and 5 years.

To further explore the clinical value of elements in ceRNAs, univariate and multivariate Cox regression analyzes were performed in the TCGA cohort. In univariate Cox analysis, LINC01122, TPD52, hsa-miR-34c-5p, hsa-miR-449a, T stage, and N stage were found to be significantly associated with the prognosis of patients with PRAD, as shown in (Fig. 9A). In addition, the results of multivariate Cox regression showed that only LINC01122, TPD52, and T stage were independent risk factors for the prognosis of prostate cancer patients (Fig. 9B). Further, we created a nomogram based on the regression analysis discussed above to give clinicians a quantitative way to forecast. The score is assigned to each prognostic factor for each patient, and a greater overall score denotes a worse prognosis for the patient. We analyzed through the R software regplot package and rms package and included the significant variables of univariate Cox regression analysis into the nomogram. Based on Total points as the dividing line, the upper part represents the factors included in the model. When the corresponding clinical information of a patient is entered, each clinical information will get the corresponding Points, and finally, the scores of these factors will be summed up, and a total score will be obtained. According to the total score, the corresponding points will be found in the Total points, and then you can look below. The probability of recurrence or death in the corresponding years of the patient was obtained, and the greater the total score, the higher the probability of death (Fig. 9C). The calibration curve gravel chart is mainly used to evaluate the accuracy of the constructed nomogram model, in which different colors represent the prediction accuracy of different years. The closer the curve is to the dotted line, the closer to the real situation, which means the more accurate the prediction result (Fig. 9D). Through the analysis of the DCA decision curve, it is found that the benefit of the nomogram in 1, 3, and 5 years is significantly better than that of other models (Fig. 9E).

### Correlation of TPD52 with drug response

Several chemotherapy medications frequently experience resistance during cancer treatment, which has a negative clinical outcome. To verify the application of TPD52 in chemotherapy, based on the pRRophetic algorithm, we predicted the IC50 of 138 drugs in high-risk and low-risk patients and found that the IC50 of all 18 drugs was significantly negatively correlated with TPD52 high expression patients, which provides guidance for patients with TPD52 high expression patients (Fig. S3).

## Discussion

PRAD is one of the high-risk cancers in men owing to its poor prognosis^20^. Therapeutic options for PRAD patients encompass surgery, androgen-deprivation therapy (ADT), radiation therapy (RT), ablative treatments, chemotherapy, and emerging immunotherapies, yet the prognosis remains unfavorable^21,22^. To identify novel therapeutic targets and improve PRAD patient outcomes, an in-depth exploration of the molecular mechanisms and disease etiology is essential, along with the discovery of promising biomarkers. Notably, competitive endogenous RNA (ceRNA) regulatory networks play pivotal roles in various human cancers^23^, including gastrointestinal cancers^24^, hepatocellular carcinoma^25^, ovarian cancer^26^, and colorectal cancer^27^. However, limited research has focused on ceRNA regulatory networks for predicting PRAD outcomes. Therefore, we embarked on an investigation to unveil a PTEN-related ceRNA triple network specific to PRAD. PTEN acts as a tumor suppressor gene involved in many cancers, specifically glioblastoma, lung cancer, breast cancer, and prostate cancer^28,29^. Intriguingly, our study involved comprehensive analyses, including copy number variation assessments, IHC, and survival analysis, which consistently affirmed our findings.

In this study, we identified 9 lncRNAs (6 upregulated and 3 downregulated), 6 miRNAs (3 upregulated and 3 downregulated), and 74 mRNAs (17 upregulated and 57 downregulated) were made up of the PRAD-associated lncRNA-miRNA-mRNA triple regulatory network. Enrichment analysis revealed that the network was mainly concentrated in “proteoglycans in cancer,” “Rap1 signaling pathway,” and “GTP binding”. Then, we discovered the significant triple regulatory network with two lncRNAs, five miRNAs, and thirteen mRNAs. The hub regulatory network was then subjected to expression analysis and survival analysis. Furthermore, we conducted the subcellular localization analysis of the two lncRNAs in the network because the interactions in the ceRNA network only exist in the cytoplasm. In conclusion, the LINC01122-hsa-miR-34c-5p/hsa-miR-449a-TPD52 ceRNA network associated with the prognosis of PRAD was obtained.

Our investigation unveiled strong correlations between miR-34c-5p and various cancers, including lung cancer^30^, colon cancer^31^, and cervical cancer^32^, and miR-449a is a potential therapeutic agent for cancer^33^. Dysregulation of TPD52, resulting from chromosomal amplification and androgen induction, has been implicated in prostate cancer development^34^. Notably, our study identified significantly higher TPD52 expression levels in PRAD compared to normal tissues, with survival analysis highlighting a link between elevated TPD52 expression and adverse outcomes. The same as TPD52, miR-34c-5p and miR-449a were overexpressed, and the high expression level of these two miRNAs suggested worse PFS prognosis in PRAD.

In the current investigation, we discovered no connection between TPD52 abnormal overexpression and copy number alterations in PRAD. In terms of study, DNA methylation is crucial for controlling the expression of genes^35,36^. Consequently, we investigated DNA methylation patterns that might be responsible for the abnormal expression of TPD52 in PRAD. We discovered that TPD52 in PRAD samples was hypomethylation compared with adjacent normal ones, which is consistent with the observed upregulation of TPD52 in PRAD combined with DNA methyltransferase analysis (DNMT1, DNMT3A and DMNT3B). Furthermore, we found a negative correlation between some methylated sites and the prognosis of PRAD patients.

According to previous studies, the prognosis of patients could be impacted by immune infiltration^37,38^. In this study, we found that several immune cell infiltration levels (B cells and macrophages) were correlated with TPD52 gene copy numbers in PRAD, and TPD52 expression was significantly correlated with tumor purity, but significantly positively correlated with infiltration levels of B cells, and CD8 + T cells in PRAD by using TIMER. Together, these results suggested that the differences induced by the LINC01122/TPD52 axis might have an effect on the modifications of the tumor immune microenvironment and the development of PRAD. To further explore the clinical value of the LINC01122/TPD52 axis, we conducted the independent prognostic analysis of pathological features and establishment of the nomogram. We found that the benefit of the nomogram in 1, 3, and 5 years is significantly better than that of other models.

For biomarkers like the LINC01122/TPD52 axis to have sufficient clinical utility, they must demonstrate high sensitivity (to correctly identify at-risk patients) and specificity (to minimize false positives). These biomarkers should ideally outperform existing diagnostic standards, such as prostate-specific antigen (PSA), which has limited specificity in distinguishing between benign and malignant conditions. Additionally, reproducibility across diverse patient cohorts and datasets is critical to ensuring reliability. Further validation is required to confirm that the LINC01122/TPD52 axis meets these standards, potentially through large-scale studies or integration into multi-parametric models that combine molecular and clinical data for enhanced predictive performance. If validated, the LINC01122/TPD52 axis could be a powerful prognostic tool to guide personalized treatment strategies in PRAD.

The ceRNA-based LINC01122/TPD52 axis that we created appears to be a viable predictive biomarker in clinical applications, however, several limitations need to be considered. First, additional experimental research into the binding affinities of lncRNAs, miRNAs, and mRNAs found in the database is necessary. Second, we need to conduct more research to better comprehend the role and mechanism of the LINC01122/TPD52 axis in PRAD. We developed a ceRNA network (LINC01122 -hsa-miR-34c-5p/hsa-miR-449a-TPD52 ceRNA) network related to the prognosis of PRAD, which is superior for understanding the relationship between lncRNA, miRNA, and mRNA. Furthermore, we discovered the ceRNA-based LINC01122/TPD52 axis can be a significant prognostic factor contributing to PRAD, and the prognostic model is helpful for examining the pathophysiology of PRAD.

## Supplementary Information

Supplementary Information.

## Data Availability

All data generated or analyzed during this study are publicly available. The TCGA data can be downloaded from the TCGA database (https://por-tal.gdc.cancer.gov/). You can contact the author of the newsletter for further inquiries.
