# Supplementary figures and images for: Comprehensive analysis of the LINC01122/TPD52 axis as a predictive biomarker in prostate adenocarcinoma
Source: Sci Rep. 2025 May 8;15:16122. doi: 10.1038/s41598-025-98219-1 (PMC12062280; doi:10.1038/s41598-025-98219-1)

Figure S1.

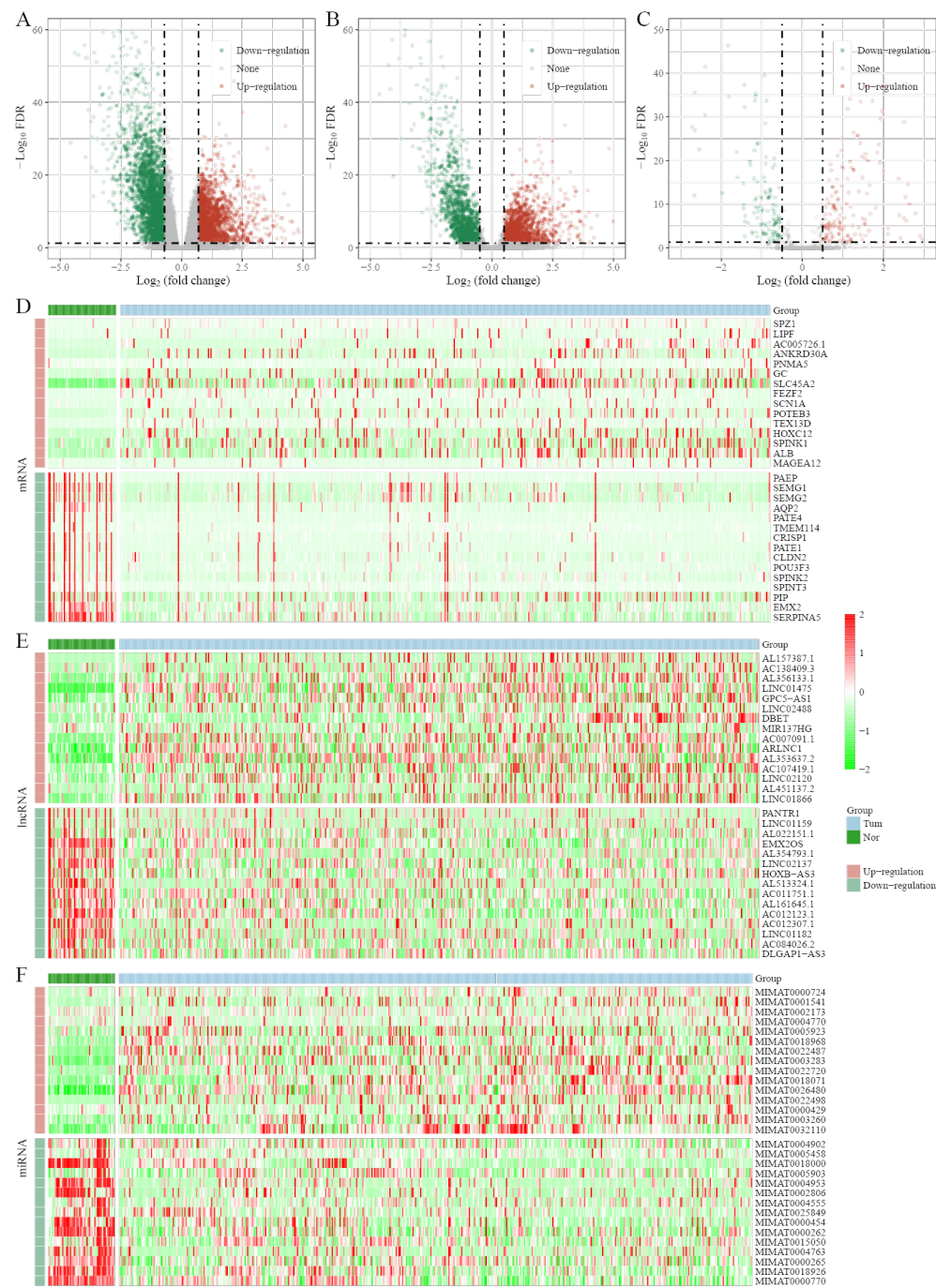

**Figure S2.**

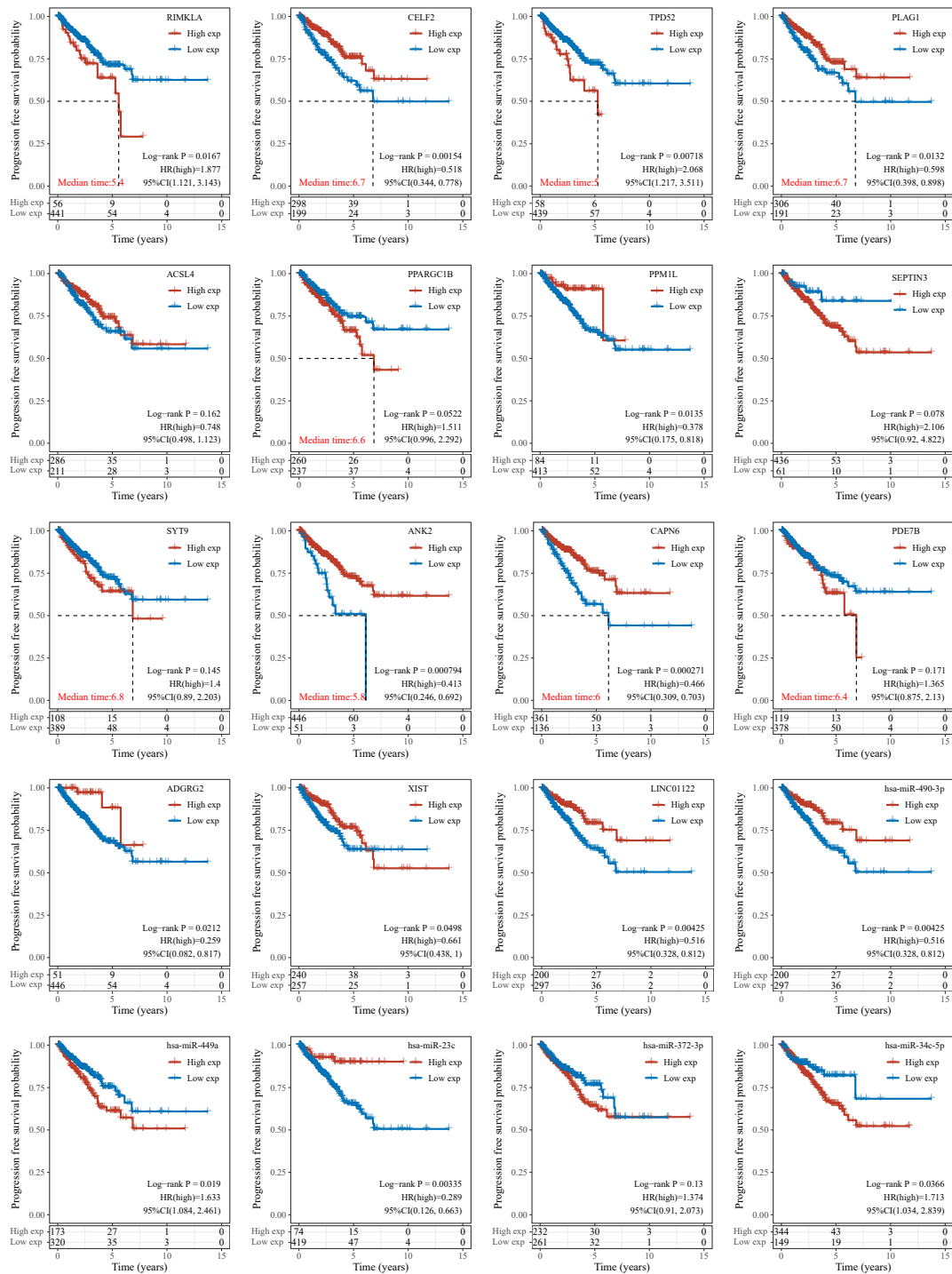

**Figure S3.**

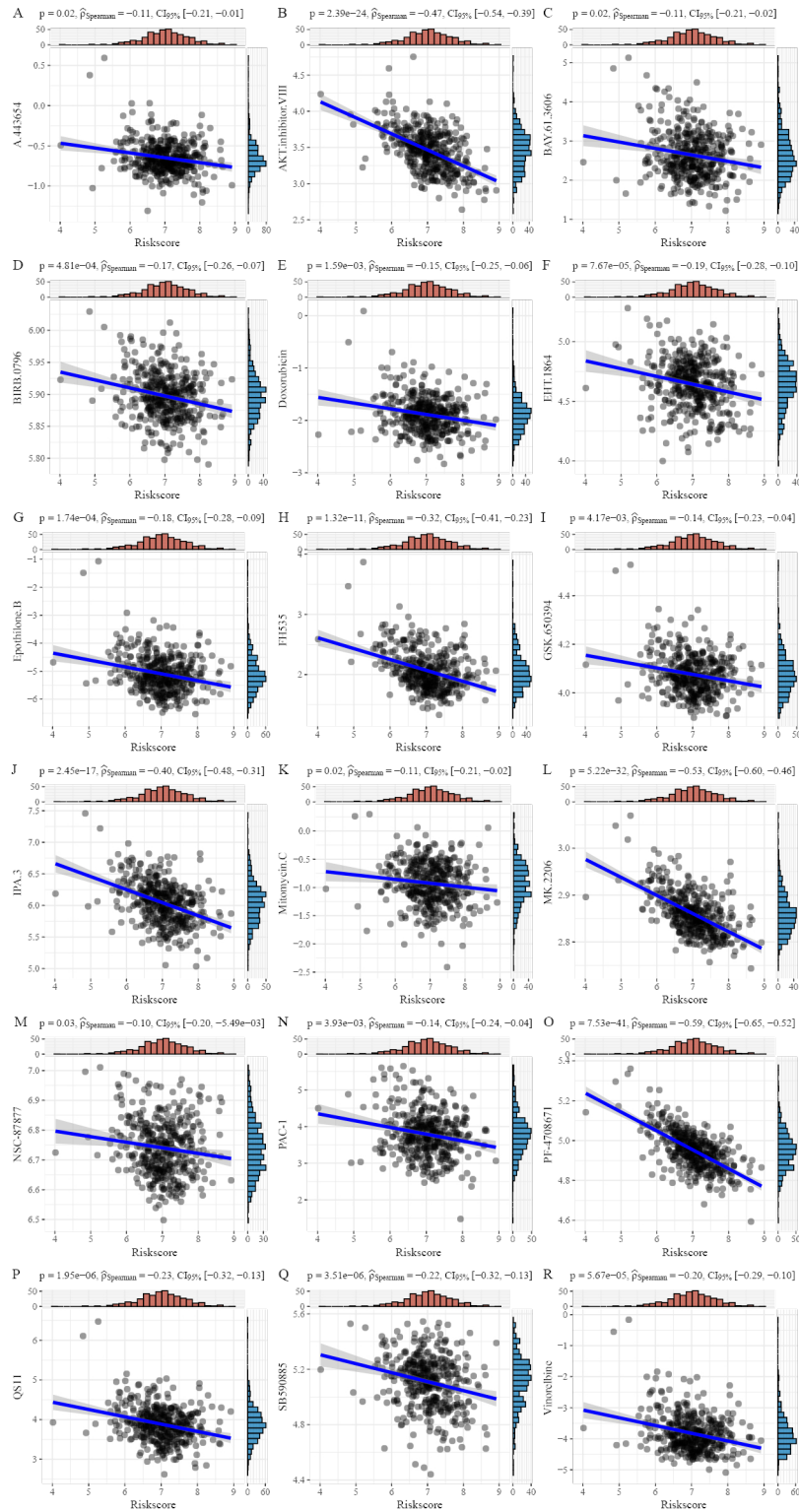

Supplement: Supplementary file 1 — Supplementary Information. [file 41598_2025_98219_MOESM1_ESM.pdf]
